# Comparison between low, moderate, and high intensity aerobic training with equalized loads on biomarkers and performance in rats

**DOI:** 10.1038/s41598-022-22958-8

**Published:** 2022-10-27

**Authors:** Carlos Dellavechia de Carvalho, Rafael Rossi Valentim, Luiz Carlos Carvalho Navegantes, Marcelo Papoti

**Affiliations:** 1 Ribeirão Preto Medical School, Department of Orthopedics and Anesthesiology, University of São Paulo, Avenida Bandeirantes, Ribeirão Preto, Monte Alegre, 3900 Brazil; 2 Ribeirão Preto Medical School, Department of Physiology, University of São Paulo, Avenida Bandeirantes, Ribeirão Preto, Monte Alegre, 3900 Brazil; 3 School of Physical Education and Sport of Ribeirão Preto, University of São Paulo, Avenida Bandeirantes, Ribeirão Preto, Monte Alegre, São Paulo 3900 Brazil

**Keywords:** Physiology, Health care

## Abstract

This study investigated the physiological and molecular responses of Wistar Hannover rats, submitted to three 5-week chronic training models, with similar training loads. Twenty-four Wistar Hanover rats were randomly divided into four groups: control (n = 6), low-intensity training (Z1; n = 6), moderate-intensity training (Z2; n = 6) and high-intensity training (Z3; n = 6). The three exercise groups performed a 5-week running training three times a week, with the same prescribed workload but the intensity and the volume were different between groups. An increase in maximal speed was observed after four weeks of training for the three groups that trained, with no difference between groups. Higher rest glycogen was also observed in the soleus muscle after training for the exercise groups compared to the control group. We also found that the Z2 group had a higher protein content of total and phosphorylated GSK3-β compared to the control group after five weeks of training. In conclusion, the present study shows that five weeks of treadmill training based on intensity zones 1, 2, and 3 improved performance and increased resting glycogen in the soleus muscle, therefore intensity modulation does not change the training program adaptation since the different program loads are equalized.

## Introduction

Physical exercise has received great attention from the scientific community, and has been used for several decades, to improve sports performance, and also promote health. Exercise is considered a non-pharmacological strategy to combat various diseases that can compromise the quality of life and longevity and that can be tackled and/or minimized by performing physical exercises, such as obesity^1^, diabetes^2^, sarcopenia^3^, Parkinson^4^, cancer^5^, cardiovascular diseases^6^, cognitive decline^7^, but recently, in the recovery of people with COVID-19^8^.

The current paradigm regarding training periodization models is built from the assumption that applied mechanical load parameters directly dictate the biological adaptations magnitude^9^. The qualitative response pattern expected from physiological stress, both acute and chronic, caused by physical training is highly consistent with the curve of the “General Adaptation Syndrome” (GAS)^10^. Based on this statement, the training program planning proposals take into account the variables that make up the training load (volume, intensity, density, complexity), because it is known that load directly influences the response magnitude and its control should be undertaken when comparing acute and chronic training models^10,11^.

Conventionally, training sessions are classified based on the relationship between physiological variables behavior (lactate, ventilation, carbon dioxide) and exercise intensity. Considering the objective of the training sessions is to improve aerobic parameters, classification is made considering three phases limited by physiological thresholds, the most common being the lactate and ventilatory thresholds. There are also classifications for chronic training models and a series of descriptive studies^12,13^ revealed that elite athletes opt for training periodization with a “polarized” distribution, i.e., a large training volume percentage (approximately 75%) performed in Zone 1 (Z1); in low intensity (> lactate threshold 1 or blood lactate concentration ([La]) < 2 mM), approximately 15% in Zone 3 (Z3); at intensities above lactate threshold 2 ([La] > 4 mM) and only a small amount of the volume (approximately 10%) in Zone 2 (Z2); and at intensities between thresholds 1 and 2 ([La] between 2 and 4 mM), despite the intensity of competitive events being close to the anaerobic threshold^12–15^. However, there is still a great deal of discussion in the literature regarding the choice of the “best” training model for outcomes related to oxidative adaptations and, consequently, in health-related parameters.

Specifically, regarding the stimuli responsible for improving aerobic performance, there are still contradictions in the literature regarding the most appropriate strategy for oxidative adaptations in skeletal muscle to occur, and consequently, performance improvement. It has recently been shown that high-intensity interval training (HIIT) can promote oxidative adaptations in skeletal muscle similar to traditional aerobic training (endurance training (ET))^16–18^.

You et al.^19^ recently investigated, in their revision, the applications of HIIT for health promotion, and they found, among other results, that HIIT can favor body composition improvements, and spend less time to achieve similar effects than continuous training; can efficiently improve cardio-metabolic capacity in overweight teenagers; can be considered as a suitable supplement of strength training method in the elderly groups. They concluded that HIIT can be effective in the health promotion domain for metabolic diseases, cardiovascular diseases, neurological diseases, and musculoskeletal diseases^19^.

In humans, training load has been determined through several methods based on the product of volume and training intensity, which are called “training impulses (TRIMP)”. These are obtained from heart rate responses^20,21^, blood lactate concentration ([La])^13,22^, and perceived exertion^23^. In animal models, the same rationale has been used^24–26^. In part, the contradictions observed in studies comparing the effects of different training models (low intensity and long volume vs high intensity and low volume) on physiological and molecular outcomes are since they use different training “loads” (i.e., training programs with different amount of volume and intensity, monitored by internal and external variables).

To understand the biomolecular adaptations resulting from physical training, the animal model provides a robust way to control independent variables^27^. Some biomolecular responses can be found in the literature on responses to different exercise intensities. It can be highlighted: increase in AMPK activity in an intensity-dependent manner after an exercise^28^ in rats^29,30^ and humans^31,32^; increase in the body's ability to oxidize glucose, mediated by increased CS, considered a marker of oxidative capacity and mitochondrial activity^33,34^. Therefore it is necessary to answer the question about how training programs should be structured and about the “dose–response” to chronic exercise^27,35^. Comparing the same workload, Teixeira-Coelho et al.^35^ have shown that overload in intensity is more effective than overload in volume at inducing performance improvements, and Forte et al.^27^ suggested that periodized training seems to be optimized to the physiological responses of rats. Finally, Carvalho et al.^36^ showed, after an acute exercise, that the muscle and hepatic glycogen depletion and recovery kinetics are not modulated by exercise intensity since the load was equalized. Since the training load is an issue, there is still no consensus in the literature on how to perform the modulation of training variables. In this context, it is not clear how the modulation of load variables alters the response in training programs if this load is equalized.

The present study aims to investigate the physiological, molecular, and performance responses of Wistar Hannover rats, submitted to three 5-week chronic training models, with different relationships between volume and intensity, but with similar training loads. Our hypothesis is that exercise stimuli will induce favorable adaptations in all tissues analyzed associated with performance improvements, with different responses among them.

## Methods

### Ethical approval

All the procedures of the present study were submitted and approved by the Committee of Ethics and Research on the Use of Animals of the School of Physical Education and Sports of Ribeirão Preto (protocol nº 2018.5.31.90.5) and experiments were performed following the specific Brazilian laws on the Bioethics in Experiments with Animals (nº 11.794/2008) and complied with the ARRIVE guidelines for the Care and Use of Laboratory Animals.

### Animals

Twenty-four Wistar Hannover rats aged 60 days were acclimatized for 15 days until they reached a mean weight of 250 g. The animals were randomly divided into four groups: control (n = 6), low-intensity training (Z1; n = 6), moderate-intensity training (Z2; n = 6) and high-intensity training (Z3; n = 6). Throughout the experiment, the animals were kept in collective cages measuring 37 × 31 × 16 cm, not exceeding four rats per cage, in a 12/12 h inverted light/dark cycle. All animals received commercial feed and water ad libitum. All experiments were carried out in the morning by the same researchers who manipulated the animals, and their weight was recorded three times a week.

### Experimental design

**Figure 1:**
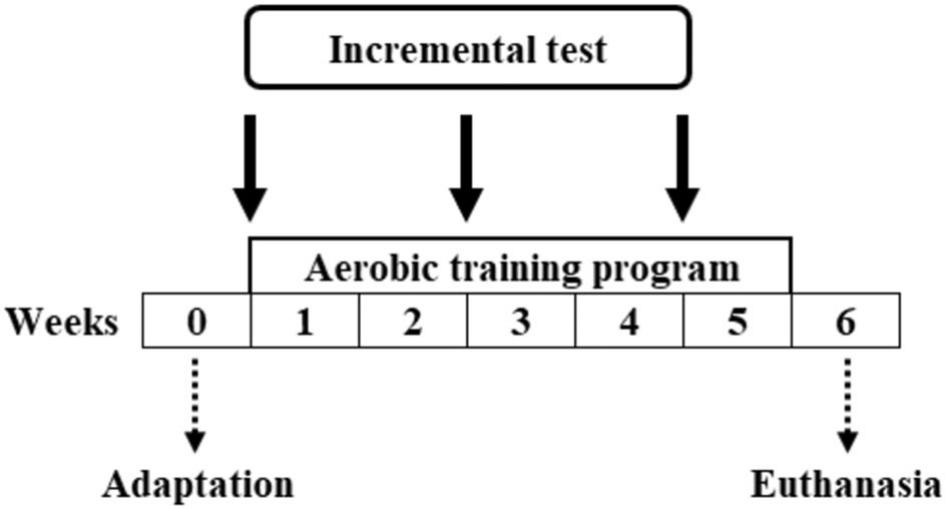
Evaluation schedule that includes adaptation to the treadmill, incremental tests, training programs and euthanasia. ↓ = indicates evaluation moments.

All procedures (training and evaluations) were performed on a treadmill and the animals were adapted to exercise for 5 days, running 10 mi·day^−1^ at a velocity between 6 and 15 m·min^−1^. All procedures were performed in the morning, between 8 and 12am. At the end of the adaptation period, the animals were subjected to an incremental test for individual training session intensities prescription. After the incremental test, the animals started the training protocols that lasted for five weeks. After 48 h following the sixth (2 weeks) and twelfth (4 weeks) training sessions, the animals again performed the incremental test to readjust the training intensity and to assess performance (Fig. 1). After the last incremental test, the animals were submitted to the last week of training. The sessions used to compose the training programs were recently standardized in our laboratory^36^.

### Incremental test (IT) and performance quantification

The recently standardized protocol was used in our laboratory^36^. In this, the animals were submitted to an incremental test with an initial speed of 8 m·min^−1^ and an increase of 3 m·min^−1^ every three minutes.

The maximal running speed (MaxS) achieved in the test was recorded and adjusted by the following Equation. ^37^:

$${\text{MaxS}} = {\text{CSV}} + \left( {\frac{{{\text{IT}}}}{{{\text{TE}}}}} \right) \times {\text{I}}$$

where CSV = last complete stage velocity; IT = last stage time performed; TE = stipulated stage time, and I = each stage intensity increase.

The rat’s performance (Pr) using a mass-dependent model^38^ was also calculated, considering the mechanical work produced, as shown in the following equation:

$${\text{Pr }} = \sum {\text{ Pr}}_{{\text{i}}} = \sum {\text{ mV}}_{{\text{i}}} {\text{T}}_{{\text{i}}} = \sum {\text{mD}}_{{\text{i}}} = {\text{mD}}$$

where Pr = rat’s performance; Pr = rat’s performance in each stage; m = body mass; V_i_ = stage velocity; T_i_ = stage running time; D_i_ = stage distance; and D = total distance covered by the rat during the test. Pr is expressed in kilogram-meters (kg.m).

### Training load quantification

The training load was assumed to be the product of duration by intensity, as an adaptation of strategies carried out with humans^39^ and also used in animals^39,40^. The intensity was assumed as the MaxS percentage and running speed, for the calculations of internal (prescribed load) and external (performed load) loads, respectively.

### Training programs

Training sessions were standardized in our previous study^36^. The animals were submitted to three 5-week running training models (Z1, Z2, and Z3) with a frequency of three times a week totaling 15 sessions. Z1 and Z2 consisted of 48 and 32 min of continuous efforts, respectively, and Z3 was an interval protocol of five 5 min and 20 s effort for 2 min and 40 s passive interval (effort/pause ratio = 2/1). The training load sessions were equalized between the sessions (2400 a. u.) (Table 1).

**Table 1:** Variables that compose the prescribed training sessions.

Groups	Volume (min)	Intensity (% MaxS)	Frequency (times.week^−1^)	Session load (a.u.)
Z1	48	50	3	2400
Z2	32	75	3	2400
Z3	5 × 5.33	90	3	2400

Z1 = low intensity training group; Z2 = moderate intensity training group; Z3 = high intensity training group; MaxS = maximal running speed; a. u. = arbitrary units.

Load adjustments were made in running speed according to the incremental test results that were applied before weeks 1, 3, and 5, to adapt the training status of the animals to the proposed load.

### Skeletal muscle extraction

Seventy-two hours after the last training session, the six animals from each group were anesthetized by intraperitoneal administration of xylazine (10 mg·kg body weight^−1^) and ketamine (100 mg·kg body weight^−1^) mixed in the same syringe. Anesthesia control was assessed by foot reflex loss^41^. Subsequently, the soleus, *extensor digitorum longus* (EDL) muscles, and liver were removed for storage and stored at −80 °C for subsequent analysis.

### Muscular glycogen assessment

To determine glycogen muscles from soleus and EDL muscles and from the liver, tissue fractions weighing between 25–23 mg were used, removed immediately after sacrifice, and digested in a bath at 100 °C in 0.5 mL of 1 N KOH for 20 min. A volume of 20 µL saturated Na_2_SO_4_ solution was added and glycogen was precipitated through two passages of 2.5 mL of hot ethanol, followed by centrifugation in 4 mL of water, and the colorimetric determination was performed in 1 mM extract, 20 µL of 80% phenol and 2.0 mL of concentrated sulfuric acid, after boiling for 15 min. Absorbance was measured using a 490 nm spectrophotometer. Known glucose solutions were used for the calibration curve^36,40,42^.

### Immunoblotting

For the protein content determination by western blot the tissues were homogenized in 50 mM RIPA buffer of Tris–HCl (pH 7.4) containing 150 mM NaCl; 1 mM EDTA; 1% Triton X-100; 0.1% SDS; 5 µg/ml aprotinin; 1 mM of PMSF; 10 mM sodium orthovanadate; 100 mM NaF; 10 mM sodium pyrophosphate; 10 uM trichostatin A; and 5 mM Nicotinamide. The homogenate was centrifuged at 17,000 g for 20 min at 4 ºC and the supernatant was separated for electrophoresis and protein measurement by the Lowry method^43^. To the homogenates, sample buffer (20% glycerol, 125 mM Tris–HCl, 4% SDS, 100 mM dithiothreitol, 0.02% bromophenol blue, pH 6.8) was added in a 1:4 ratio. Before electrophoresis, samples were boiled at 100 ºC for 5 min for protein denaturation. Following electrophoresis, proteins were transferred from the gel to the nitrocellulose membrane using buffer (48 mM Tris, 39 mM glycine, 10% SDS, and 20% methanol) in a semi-dry system for 30 min under the fixed voltage of 20 V, at room temperature (BioRad Trans-Blot SD Cell, USA)^44^. After the transfer was completed, the membrane was subjected to immunoblot. The membrane was blocked by incubation for 1 h, under agitation, at room temperature in a 5% skimmed milk powder solution, diluted in TBS-T solution (20 mM Tris–HCl; 160 mM NaCl and 0.1% Tween 20). The membrane was then incubated overnight at 4 ℃ under agitation with specific primary antibodies (anti-p-AMPKα, anti-citrate synthase, anti-GSK3β, anti-pGSK3β, anti-OXPHOS, and anti-β-actin) diluted in TBS-T containing 2.5% bovine albumin and 0.01% sodium azide. The following day, incubation with the secondary antibody was performed for 1 h at room temperature. The membranes were revealed in a photo-documenter (BioRad) using an amplified chemiluminescence kit (ECL, Amersham). The revealed bands were photographed and quantified by densitometry using Image Lab software (BioRad). After densitometric quantification of proteins, the results were expressed by the relationship between the densities obtained for the protein of interest and the normalizing protein, used as application control, as indicated in each result.

### Statistical analyzes

Data are expressed as mean ± standard deviation, except for incremental test data, which are expressed as median and interquartile range. Data normality was tested using the Shapiro–Wilk test. The comparisons between groups were performed using One-Way Analysis of Variance followed, when necessary, by Tukey's Post-hoc test), except for incremental test data, in which, it was used the Friedman test (Statistica 7.0® Statsoft, Tulsa, OK. In all cases, the level of significance was set at *p* < 0.05.

## Results

**Figure 2:**
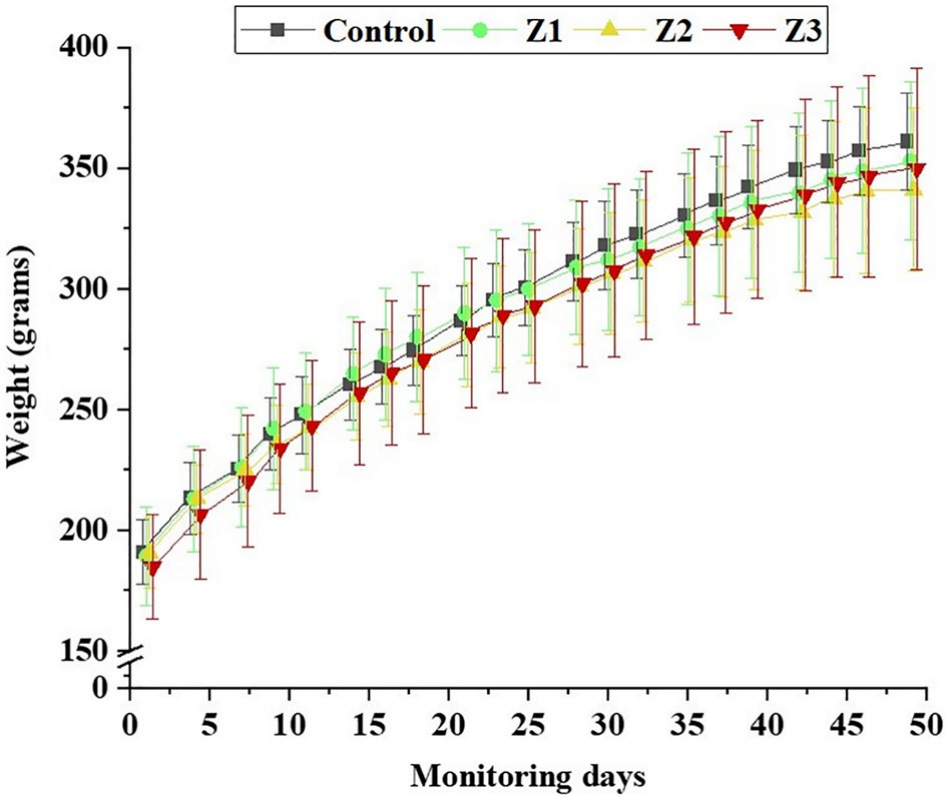
Weight of rats throughout the evaluation period. Z1 = low intensity training group; Z2 = moderate intensity training group; Z3 = high intensity training group.

The weights of rats throughout the monitoring were not different between the groups (*p* > 0.05) (Fig. 2).

**Figure 3:**
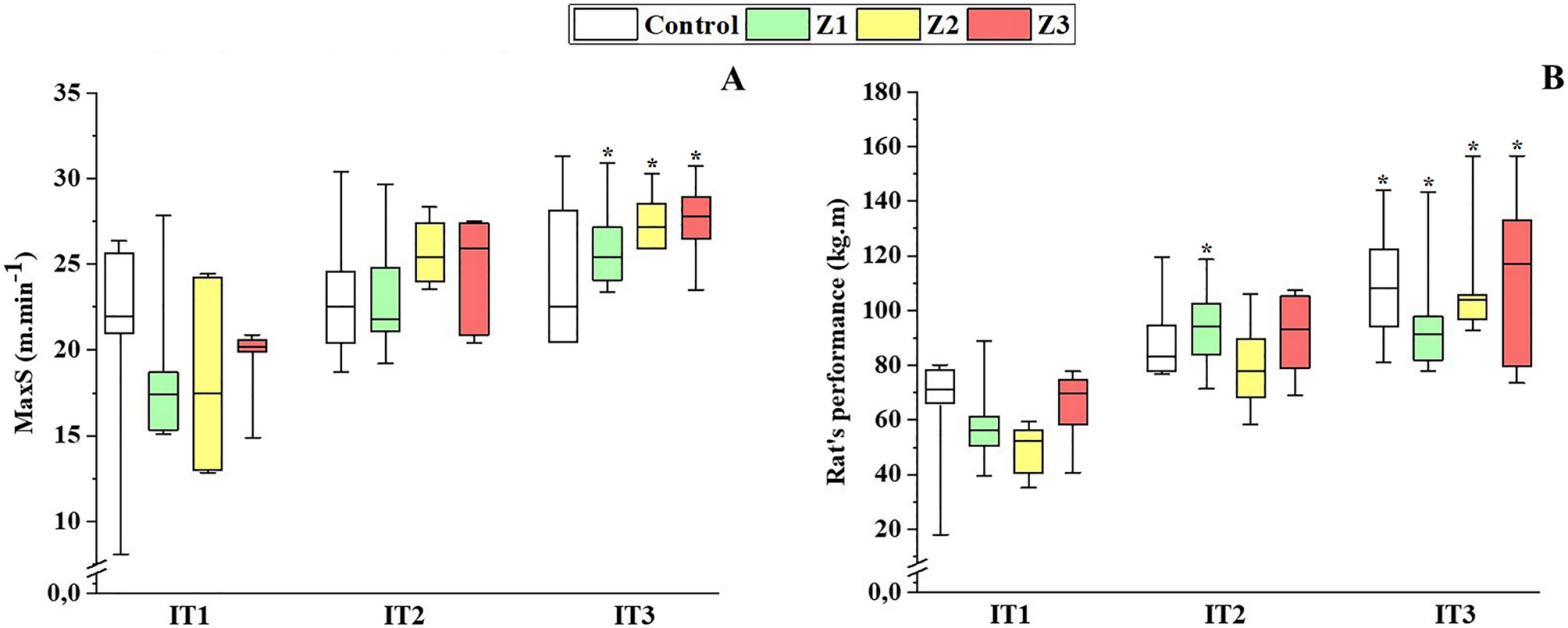
Effect of four weeks of running training on the maximal speed (MaxS) achieved in the incremental test (IT) (**A**) and rats’ performance quantified (**B**). Z1 = low intensity training group; Z2 = moderate intensity training group; Z3 = high intensity training group; IT = incremental test; * = statistically significant difference compared to IT1.

All animals (n = 18) submitted to the training models completed all the proposed training sessions. Although the velocity was different between groups, there was no difference in the animals' session external load (*p* > 0.376) (Table 2). The speed was adjusted based on the results of the incremental tests, which means that if performance increased, training speed should also increase; thus, the training load increased and in the 5th week the animals trained with a significantly higher load than in the first two weeks (*p* < 0.029) (Fig. 3A). The control group showed no difference in MaxS between the three incremental tests (*p* = 0.311) (Fig. 3A). MaxS was higher in IT3 compared to IT1, for groups Z1 (*p* = 0.002), Z2 (*p* = 0.012) and Z3 (*p* = 0.028) (Fig. 3A). The rats’ performance increased for all groups after four weeks, but only for Z1 after two weeks (*p* > 0.05) (Fig. 3B).

**Table 2:** Variables that compose the accomplished training sessions.

Groups	Volume (min)	Velocity (m.min^−1^)	Frequency (times.week^−1^)	Session load (a. u.)	Training load (a. u.)
Z1	48	10.95 ± 1.91*	3	525.39 ± 91.75	7880.80 ± 1376.31
Z2	32	17.19 ± 2.11*	3	550.08 ± 67.58	8251.20 ± 1013.70
Z3	5 × 5.33	20.84 ± 1.79*	3	548.91 ± 47.27	8233.58 ± 709.02

Z1 = low intensity training group; Z2 = moderate intensity training group; Z3 = high intensity training group; MaxS = maximal running speed; a. u. = arbitrary units. * = statistically significant difference compared to the other two groups.

**Figure 4:**
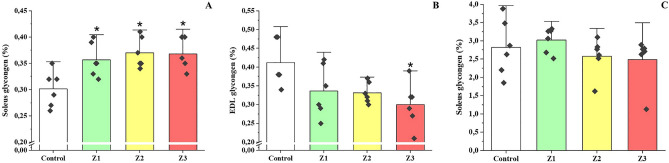
Glycogen concentration expressed in tissue percentage (%) in the soleus (**A**), EDL (**B**), and liver (**C**) muscles of the four experimental groups after five weeks of intervention. Z1 = low intensity training group; Z2 = training group at moderate intensity; Z3 = high intensity training group; * = statistically significant difference for the control group.

In the soleus muscle, the glycogen concentration ([Glic]) was higher for the three groups submitted to training programs (Z1, Z2, and Z3) compared to the control group (*p* < 0.034) (Fig. 4A). In the EDL muscle, the [Glic] were lower only for the Z3 group compared to the control group (*p* = 0.021) (Fig. 4B). No significant differences were found in hepatic glycogen between experimental groups (*p* > 0.05) (Fig. 4C).

**Figure 5:**
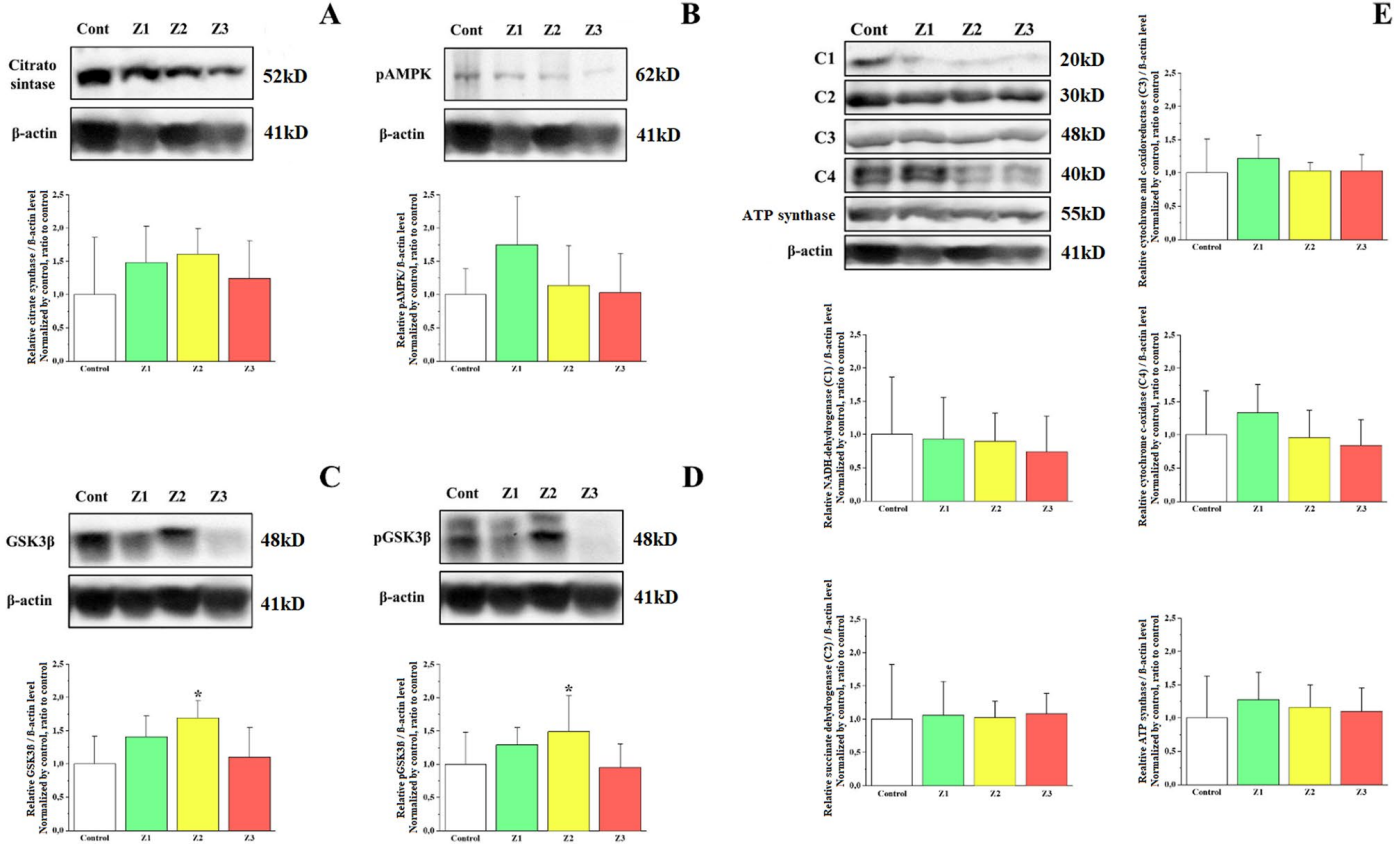
Protein content of CS (**A**), phosphorylated AMPK (**B**), GSK3β (total and phosphorylated protein (**C** and **D**), and oxidative phosphorylation complexes (OXPHOS; E) in the soleus muscle of the four experimental groups after five weeks of intervention. C1 = NADH-dehydrogenase; C2 = succinate dehydrogenase; C3 = cytochrome and c-oxidoreductase; C4 = cytochrome c-oxidase; Cont = control group; Z1 = low intensity training group; Z2 = moderate intensity training group; Z3 = high intensity training group; * = statistically significant difference for the control group.

No significant differences were found in the protein content of citrate synthase (CS) (Fig. 5A), pAMPK (Fig. 5B), and oxidative phosphorylation complexes (OXPHOS) (Fig. 5E) (*p* > 0.05). The protein content of GSK3β (Fig. 5C) and pGSK3β (Fig. 5D) were higher only in the Z2 group compared to the control group (*p* < 0.05).

## Discussion

In the present study, we compared the biomolecular adaptations and performance of rats submitted to three 5-week treadmill training protocols, in Zones 1, 2, and 3 of intensity, respectively, but with similar loads. An increase in MaxS was observed after four weeks of training for the three groups that trained, with no difference between groups. Higher [Glic] were also observed in the soleus muscle after five weeks of training for the three groups who trained compared to the control group. The Z2 group had a higher protein content of total and phosphorylated GSK3-β compared to the control group after five weeks of training. It also found an increase in rats’ performance for all groups including the control, and we believe this could be explained by the increase in animal body weights throughout the experiment.

Traditionally, the prescription of training aimed at improving health-related aspects has aerobic characteristics, usually performed in moderate intensity^45–47^. The actual exercise recommendations for health (American College of Sports Medicine) states that all healthy adults aged 18–65 years should participate in moderate-intensity aerobic physical activity for a minimum of 30 min 5 days per week or vigorous-intensity aerobic activity for a minimum of 20 min on 3 days per week) focus in aerobic exercise and the minimum weekly exercise time recommended. But our results show no difference in animal performance when comparing different training models, with the same training loads, which is important for the debate on training program planning in athletes (both professional and amateur).

Recently, Teixeira-Coelho et al.^35^ showed in Wistar rats, that the load progression carried out by increasing the intensity was more effective, compared to the increase by volume, to increase running performance after 8 weeks of intervention, keeping the loads equal. In the present study, the load progression occurred from the fourth week onwards, with an increase in absolute intensity, resulting in improved performance.

Confirming our initial hypothesis and corroborating with Milanović, Sporiš, and Weston^48^, we observed increases in the performance index (MaxS) for all animals submitted to training protocols in comparison to the evaluation before the intervention, with no changes in the control group. The training protocols were prescribed containing the same external load, i.e., the exercise “dose” to which the animals were submitted was the same for Z1, Z2, and Z3, so it is possible to consider that performance increases are dependent on the load which is imposed, as we found no difference in MaxS between the groups that trained.

When comparing training models, Milanović, Sporiš, and Weston^48^ showed utilizing a meta-analysis, a possible small chance of considering high-intensity training better than moderate-intensity training for increases in maximum oxygen consumption. The intensity comprising Zone 2 (delimited by the lactate and ventilatory metabolic thresholds) represents the highest exercise intensities that can be maintained for long periods in a stable state, making it attractive to compose a training program^13^. For decades it was assumed that the “ideal” intensity for aerobic capacity development and consequently performance improvement in medium and long duration tests, is the anaerobic threshold intensity, i.e., intensities close to the maximal lactate steady state intensity and, for sedentary and poorly trained, this seems to be true^49–51^, but recent descriptive studies indicate that for high-performance athletes the polarized model seems to be more effective. In the present study, we found no differences in animal performance when comparing training models.

It was believed that the best training model aiming at improvements related to aerobic metabolism was moderate-intensity training (close to the anaerobic threshold), and Ferreira et al.^52^ observed aerobic capacity improvements in mice submitted to training based on maximal lactate steady state, but Carvalho et al.^53^ also testing the same protocol in mice, did not find performance increases.

De Araujo et al.^54^ showed an increase in the aerobic capacity of rats after four weeks of running training on a treadmill, consisting of continuous exercise sessions. This corroborates the findings of the present study since the session used was similar to our Z2 group, maintained after the eight weeks that made up this protocol, and the same authors did not observe a difference in the analyzed parameters of another group of animals submitted to an interval protocol with intensity variation during the session^54^. Thus, the present study shows the importance of considering the exercise load to promote adaptations that are compatible with the applied stimulus.

There is evidence in the literature showing that volume and intensity play an important role in adaptations to training programs^55^. But, as the training load measured by the product of these two variables can be considered as a way of quantifying the training “doses”^56^, it seems logical to consider that the adaptations would be the same when the loads are the same for different protocols such as in the present study.

Glycogen represents the primary fuel source for maintaining adenosine triphosphate (ATP) homeostasis, and therefore, for energy availability during moderate to intense exercise sessions^57–60^, and muscle glycogen levels represent a determining factor for performance^60^. Corroborating with the GAS, after an exercise it is possible to observe a decrease in the glycogen stores (depletion), followed by an increase in this substrate during the period of recovery from the effort, being able to surpass the previous levels^58,59,61^. Thus, it is to be expected according to the GAS^62^, that greater [Glic] will be found after the recovery of greater substrate depletion.

Forte et al^25^ recently showed glycogen depletion after an acute swimming exercise, in the gluteus muscle with no difference between groups, that performed the exercise in different intensities, but with equal loads. In previous studies, we have seen that training sessions performed in different zones of intensity, but with similar loads, have no difference in muscle and liver glycogen recovery depletion kinetics, so it is logical to hypothesize that the training programs used in the present study present similar adaptation about the [Glic] after five weeks of training, which was true for the soleus muscle, which showed higher concentrations of this substrate compared to the control group, and for the liver which showed no difference between the groups^36^.

Regarding the comparison between training models, Burgomaster et al.^18^, when comparing the effects of six weeks of HIIT with ET, found similar increases in peroxisome proliferator-activated gamma coactivator receptor 1-alpha (PGC1-α), pyruvate dehydrogenase kinase (PDK), CS and peak oxygen uptake. Together the results presented demonstrated the possibility of HIIT, even based on predominantly anaerobic training, to improve oxidative capacity, maximum aerobic power, and the performance of active people, however, the workload performed was not the same between groups of this study.

For protein analysis, we chose to use the soleus muscle (predominantly oxidative muscle), considering that this muscle showed differences in [Glic] between the groups submitted to training programs and the control group, so we assume that we could associate this adaptation with the signaling pathways of the analyzed proteins (pAMPK, CS, GSK3-β, pGSK3-β, and OXPHOS complexes)^63–65^.

Regarding the regulatory mechanisms responsible for aerobic adaptations resulting from physical training that causes molecular modulation of mRNA and protein levels^27^, and depending on the intensity performed, different mechanisms are activated. High volume exercise causes a prolonged increase in intramuscular calcium, leading to calcium-calmodulin kinase activation, while high-intensity exercise leads to adenosine triphosphate concentrations reduction, with concomitant adenosine monophosphate increase, activating AMP-activated protein kinase (AMPK)^66^. Both mechanisms lead to peroxisome proliferator-activated receptor-γ coactivator-1α (PGC-1 α) activation, described as a “master switch” for mitochondrial biogenesis^66^.

After a training period, an increase in AMPK, CS, and OXPHOS complexes content is expected^67^. Rasmussen, Hancock, and Winder^30^ found in an animal model, that an increase in AMPK content after two moderate and high-intensity exercise models, respectively, concomitant with decreases in glycogen content and the enzymes malonyl-CoA and acetyl-CoA carboxylase. Thus, evidence indicates that AMPK has its activity increased after physical exercise^68^ and regulates levels of enzymes related to increased fat oxidation to maintain glucose homeostasis^30,64,69–71^. De Souza Cordeiro et al^72^ showed that a 10-week running training increased CS activity in the soleus muscle of Wistar rats. Fiorenza et al.^73^ observed significant increases in complexes I, II, III, and IV after six weeks of high-intensity training in a healthy male group, in comparison with the moment before the intervention and control group. In the present experiment, we did not observe differences in either CS or OXPHOS complexes between the groups, indicating that the five weeks of training did not alter the protein content of these complexes in the soleus muscle, indicating that there was no perceptible change in mitochondrial activity in this specific muscle.

When phosphorylated, GSK3-β becomes inactive, and has already been demonstrated by Nikoulina et al.^74^ that its inhibition leads to an increase in glycogen synthase enzyme activation, and consequently an increase in glycogen content^75^. Thus, the largest [Glic] observed in the groups that performed the training protocols, only the Z2 group can be explained by the concomitant protein increase of GSK3-β and pGSK3-β for this group.

It would be expected that both glycogen supercompensation and performance improvement would be accompanied by increased activation of energy pathways signaled by the proteins analyzed in the present study. Based on the results presented above, it can be speculated that the protein signaling would have already decreased by the time euthanasia took place (72 h after the last training session). Another hypothesis that should be considered to explain these results is the increase in AMPK activity magnitude is greater in muscle with a predominance of fast twitch fibers compared to slow twitch fibers (soleus muscle, in the case of the present study)^67^.

As a limitation, in the present study, the intervention time factor may have directly influenced the results, as it was probably an insufficient time to generate perceptual adaptations at the biomolecular level. Thus, these findings suggest that a longer period of intervention is applied, so that adaptations occur. Also, it was not used a more accurate evaluation method, such as lactate threshold, because in some of our previous studies, with unpublished data, we were not able to standardize an evaluation protocol for running in rats, using lactate, but the Vmax is commonly used variable to prescribe the intensities in training programs for animal model. Finally, training periodization is an extremely complex field with many variables to be understood, and more studies are necessary to understand the role of exercise doses in physiological adaptations.

As clinical implications, both for high performance and for health promotion, the present study points out the importance of considering the training load (that is, the exercise dose) as a fundamental variable to be controlled, which will have a great influence. adaptations resulting from any physical training program. That is, it will be possible to expect similar adaptations from training programs with different volume and intensity ratios, as long as the load is equalized.

In conclusion, the present study shows that five weeks of treadmill training based on intensity zones 1, 2, and 3 improved performance and increased the [Glic] in the soleus muscle, therefore the intensity modulation does not change the training program adaptation since the different program loads are equalized.

## Supplementary Information

Supplementary Information 1.

Supplementary Information 2.

Supplementary Information 3.

Supplementary Information 4.

Supplementary Information 5.

Supplementary Information 6.

Supplementary Information 7.

## Data Availability

All data will be available upon a plausible request to the authors.
