# Supplementary figures and images for: Comparison between low, moderate, and high intensity aerobic training with equalized loads on biomarkers and performance in rats
Source: Sci Rep. 2022 Oct 27;12:18047. doi: 10.1038/s41598-022-22958-8 (PMC9610360; doi:10.1038/s41598-022-22958-8)

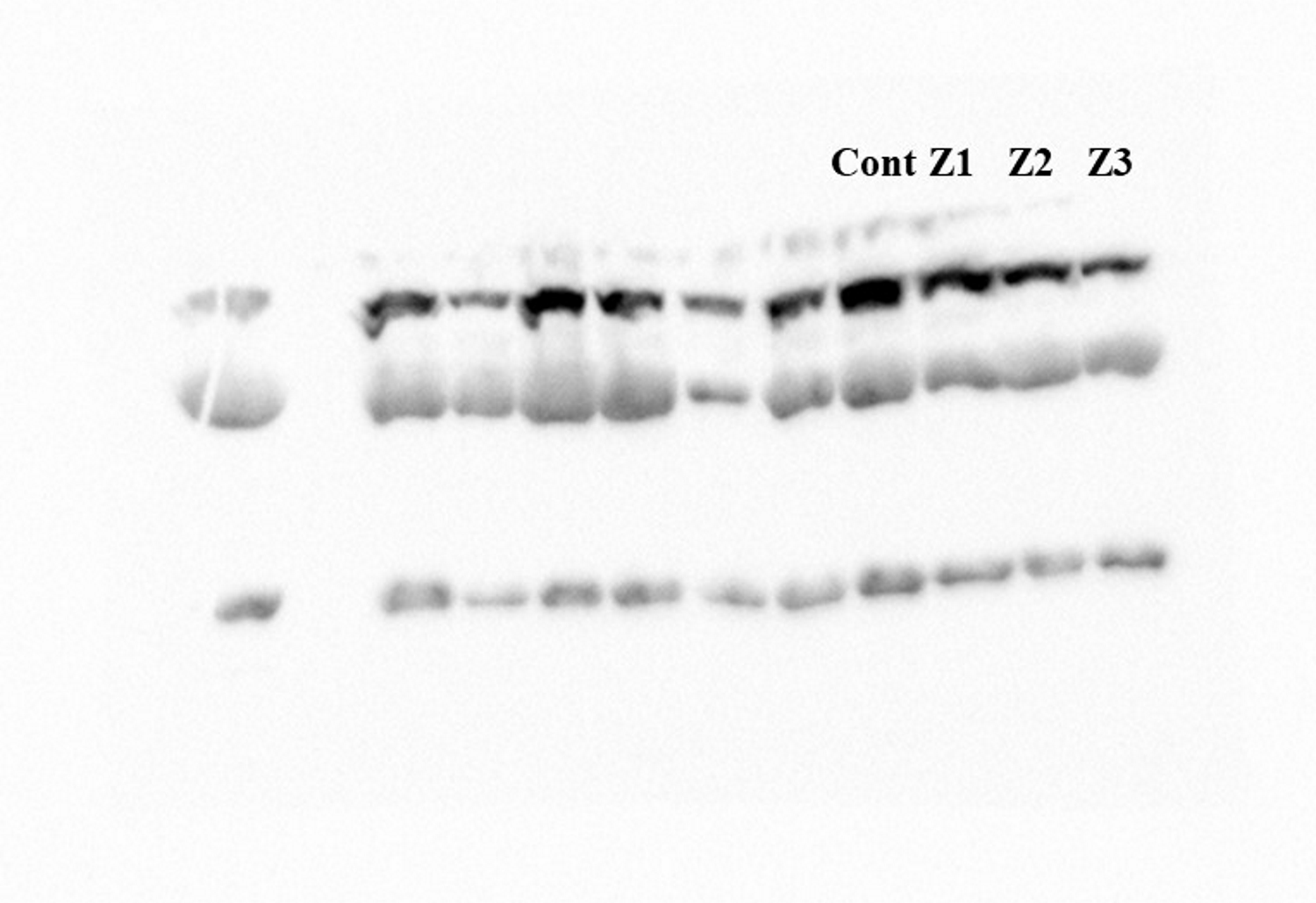

Supplement: Supplementary file 2 — Supplementary Information 2. [file 41598_2022_22958_MOESM2_ESM.png]

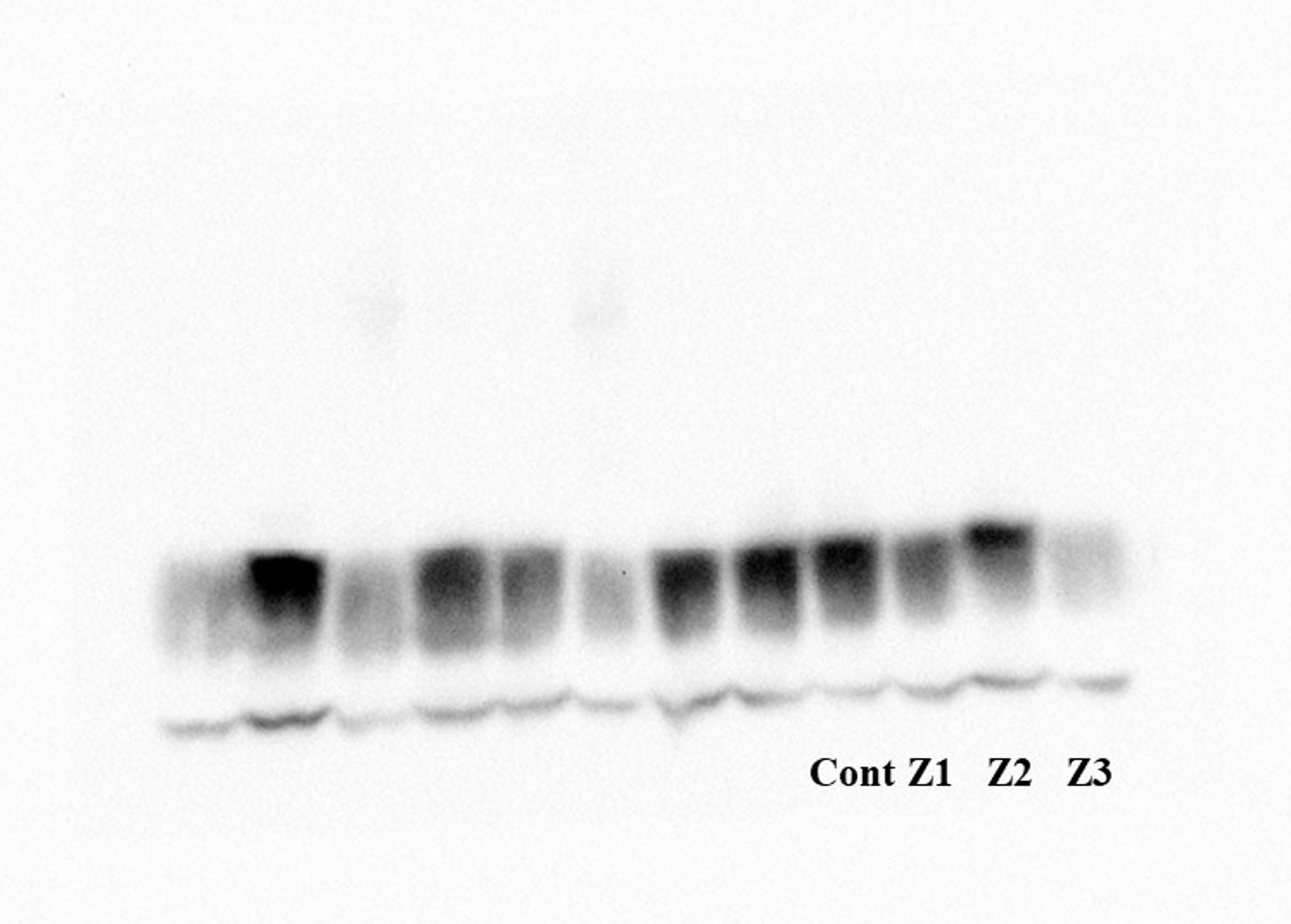

Supplement: Supplementary file 3 — Supplementary Information 3. [file 41598_2022_22958_MOESM3_ESM.png]

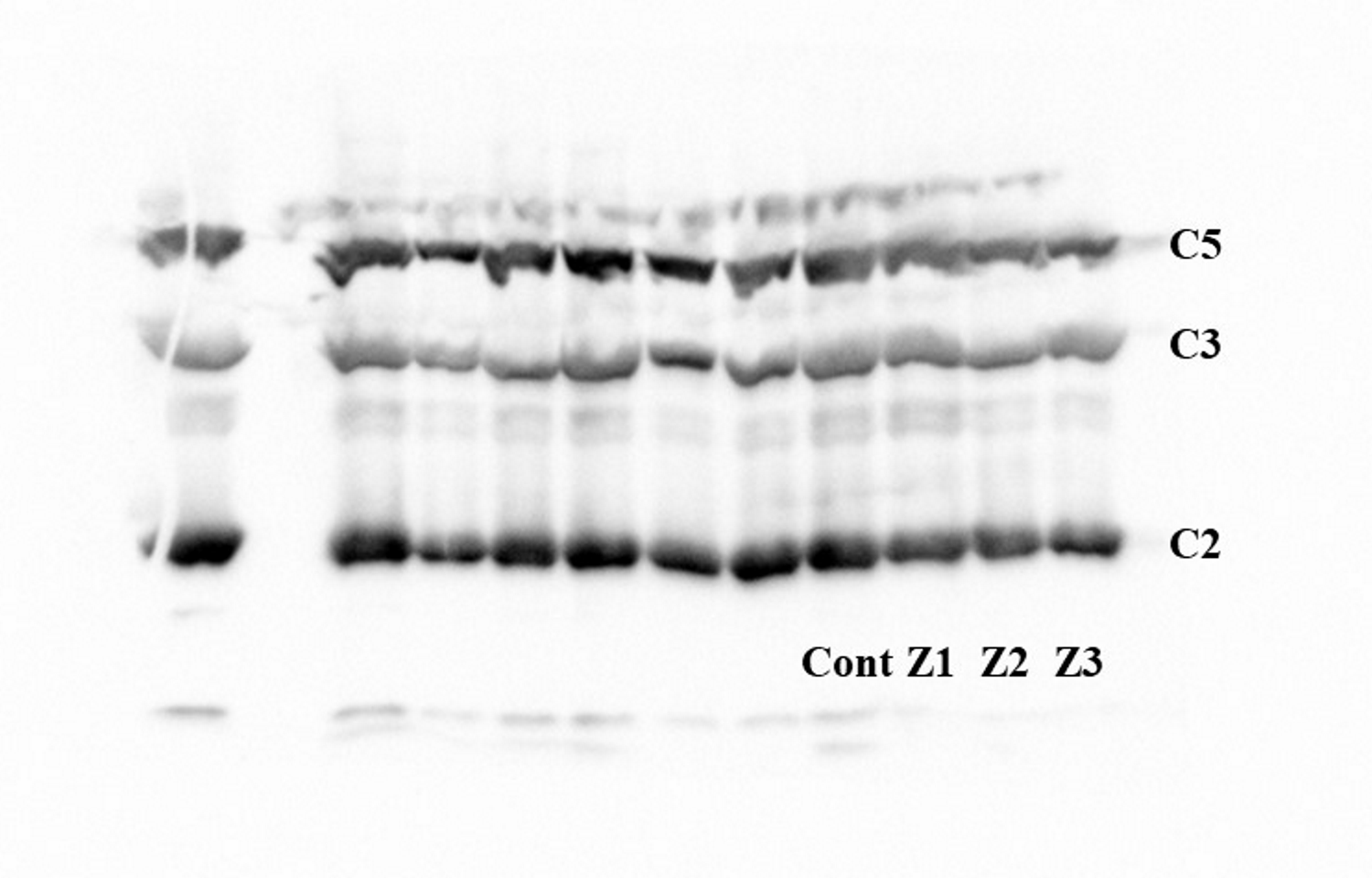

Supplement: Supplementary file 4 — Supplementary Information 4. [file 41598_2022_22958_MOESM4_ESM.png]

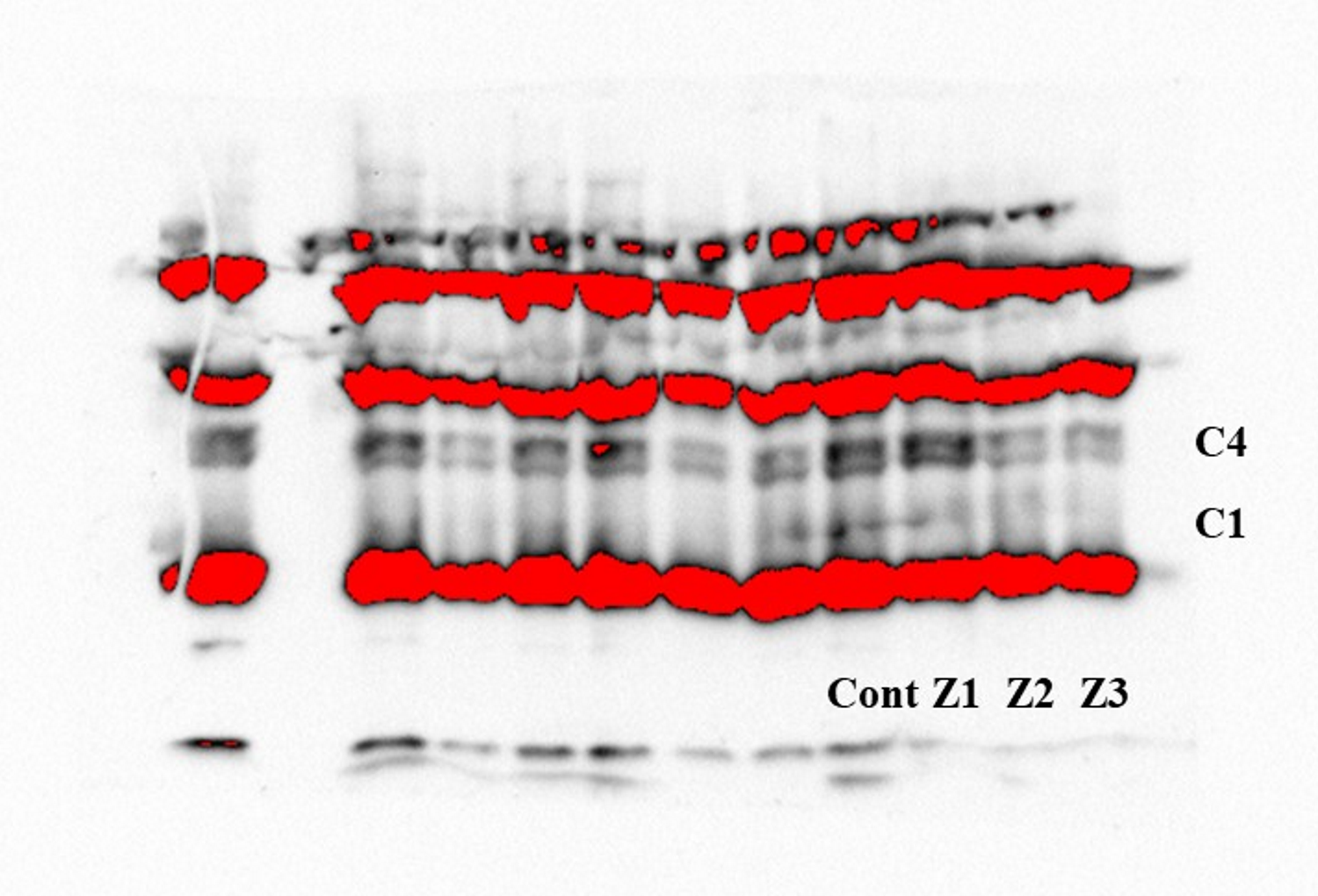

Supplement: Supplementary file 5 — Supplementary Information 5. [file 41598_2022_22958_MOESM5_ESM.png]

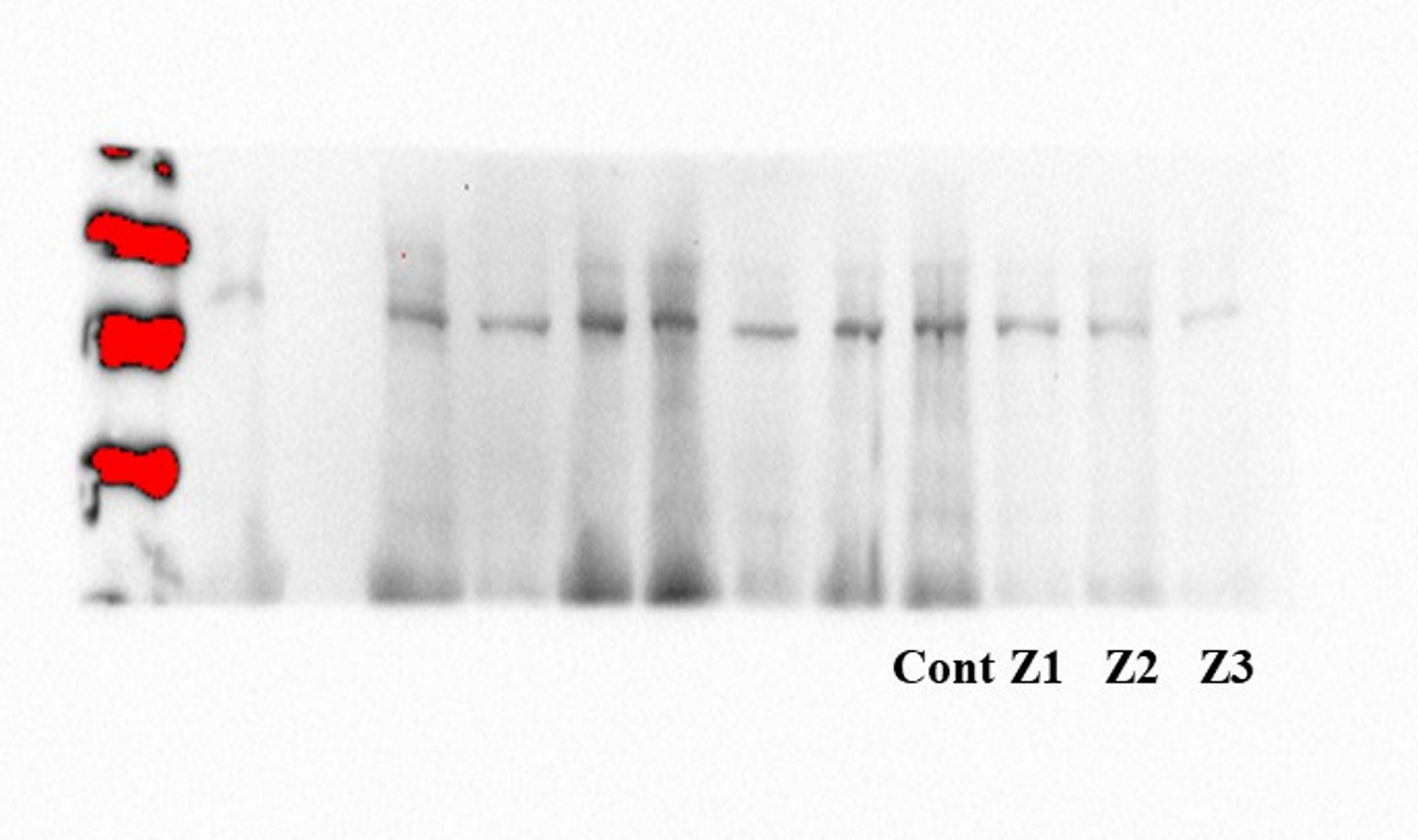

Supplement: Supplementary file 6 — Supplementary Information 6. [file 41598_2022_22958_MOESM6_ESM.png]

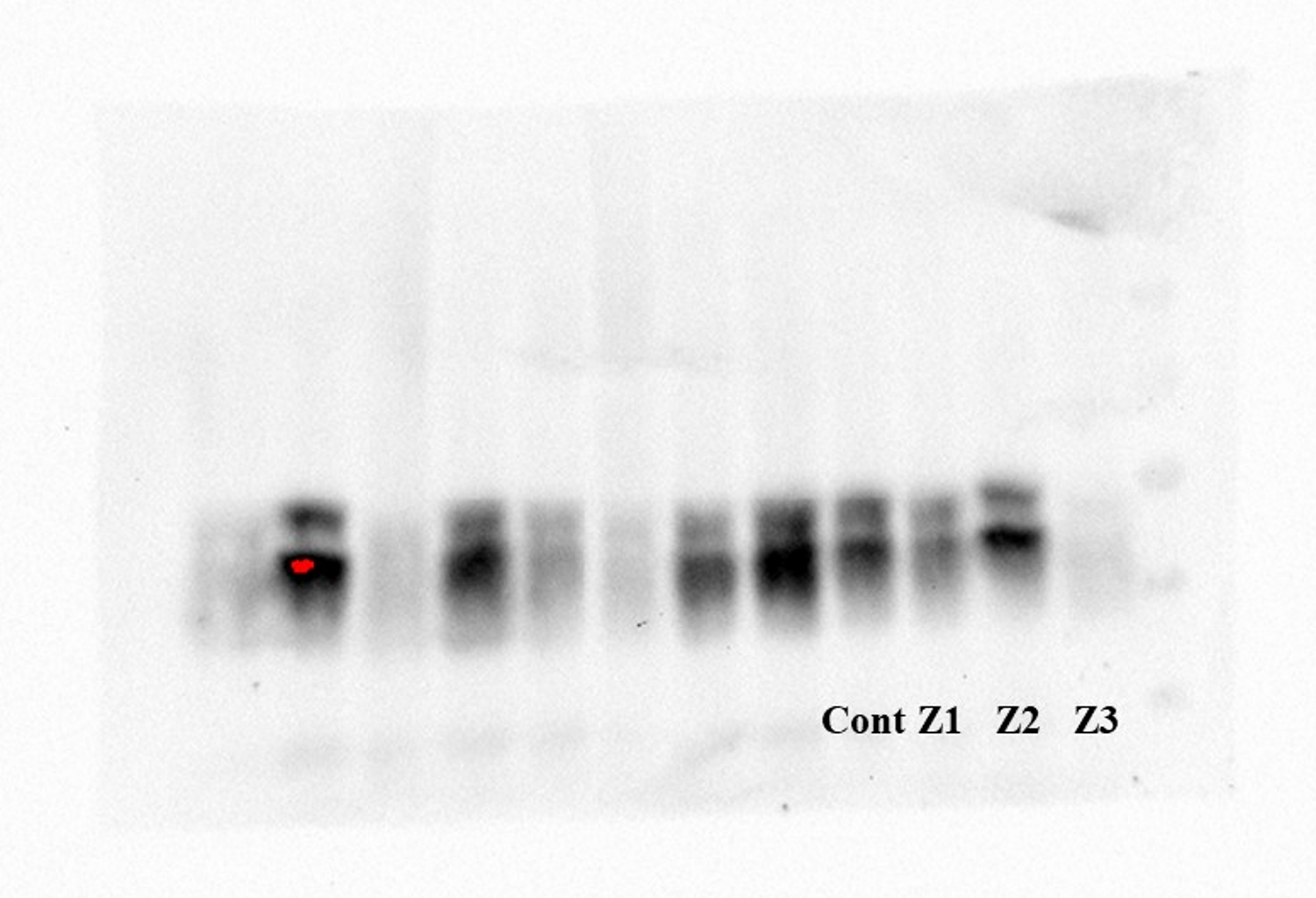

Supplement: Supplementary file 7 — Supplementary Information 7. [file 41598_2022_22958_MOESM7_ESM.png]
